# Pitfalls of haplotype phasing from amplicon-based long-read sequencing

**DOI:** 10.1038/srep21746

**Published:** 2016-02-17

**Authors:** Thomas W. Laver, Richard C. Caswell, Karen A. Moore, Jeremie Poschmann, Matthew B. Johnson, Martina M. Owens, Sian Ellard, Konrad H. Paszkiewicz, Michael N. Weedon

**Affiliations:** 1 University of Exeter Medical School, RILD Building, Barrack Road, Exeter EX2 5DW, UK; 2 Wellcome Trust Biomedical Informatics Hub, Geoffrey Pope Building, Stocker Road, University of Exeter, Exeter EX4 4QD, UK; 3 Department of Molecular Genetics, RILD Level 3, Royal Devon and Exeter NHS Foundation Trust, Barrack Road, Exeter, UK

## Abstract

The long-read sequencers from Pacific Bioscience (PacBio) and Oxford Nanopore Technologies (ONT) offer the opportunity to phase mutations multiple kilobases apart directly from sequencing reads. In this study, we used long-range PCR with ONT and PacBio sequencing to phase two variants 9 kb apart in the *RET* gene. We also re-analysed data from a recent paper which had apparently successfully used ONT to phase clinically important haplotypes at the *CYP2D6* and HLA loci. From these analyses, we demonstrate PCR-chimera formation during PCR amplification and reference alignment bias are pitfalls that need to be considered when attempting to phase variants using amplicon-based long-read sequencing technologies. These methodological pitfalls need to be avoided if the opportunities provided by long-read sequencers are to be fully exploited.

Accurately determining genotype phase is important in many aspects of genetics. For example, in pharmacogenetics^1^, transplant HLA typing^2^ and disease association mapping^3^. Until recently, haplotype phasing has generally relied on parental genotypes or statistical phasing based on allele frequency patterns within the population. Next generation sequencing technologies are often not able to phase variants that are more than a few hundred base pairs apart because of short read lengths. Recent developments in long-read single molecule sequencing technologies such as the Oxford Nanopore Technologies (ONT) and Pacific Biosciences (PacBio) sequencing systems now promise efficient and accurate haplotype phasing over multiple kilobase distances. For example, Ammar *et al.*^4^ recently used the Minion sequencer from ONT to apparently phase variants at the *CYP2D6* and HLA loci into clinically important haplotypes.

Hirschsprung disease is a congenital abnormality characterised by complete or partial intestinal obstruction resulting from an absence of neuronal ganglion cells in the intestinal tract. The extent of the aganglionosis is classified as short or long-segment disease or total colonic aganglionosis. Mutations in the *RET* gene account for a high proportion of Hirschsprung cases and are more frequent in patients with long-segment disease or total colonic aganglionosis^5^. We identified two heterozygous coding variants in the *RET* gene in a 5-month old female with total colonic aganglionosis: a *de novo* mutation, p.Arg418Ter (Chr10(GRCh38):43109219C > T), and a previously reported variant, p.Leu56Met (Chr10(GRCh38):43100551)^6^. The clinical significance of the p.Leu56Met variant is uncertain. It is possible that the p.Leu56Met is a modifier of the disease phenotype observed in our patient or is a benign polymorphism. If both variants occur on the same chromosome (in cis) then this would indicate that the p.Leu56Met variant was not contributing to the phenotype as the truncated transcript would be subjected to nonsense-medicated decay.

p.R418X and p.L56 M occur 9 kb apart and in this study we used long-range PCR amplification and ONT and PacBio sequencing to phase these variants. We also re-analysed the sequencing data from Ammar *et al.*^4^. From these analyses, we demonstrate PCR-chimera formation during PCR amplification and reference alignment bias are major pitfalls that need to be considered when attempting to phase variants using amplicon-based long-read sequencing technologies.

## Results

### Phasing two *RET* variants in a single individual using amplicon-based Oxford Nanopore Technologies sequencing produces three high frequency haplotypes

**Figure 1:**
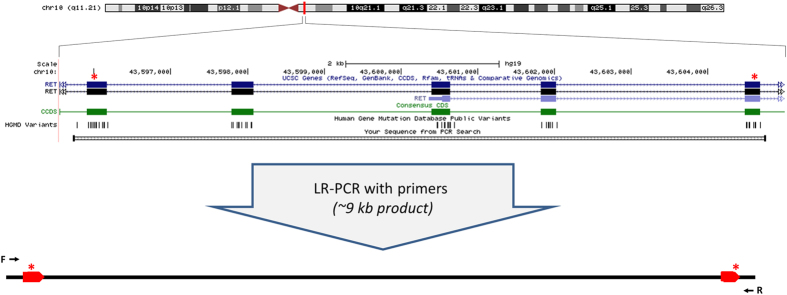
Graphical representation of the *RET* gene and the targeted mutations, including primer positions. Image adapted from UCSC genome browser^24^.

We attempted to phase two variants that are 9 kb apart in the *RET* gene in a patient with Hirschsprung disease (Fig. 1). After long-range PCR amplification we sequenced the amplicon on an ONT MinION and aligned using LAST^7^. We counted the number of aligned reads supporting each of the four possible haplotypes formed from the two variants. Only two haplotypes are expected, but all four haplotypes were present. The high error rate of the ONT system (see Table 1, error rate consistent with published literature^8^,^9^) means that we would expect to see some low-level evidence of a third and fourth haplotype; however, three of the four haplotypes were observed at high frequency (Table 2A). PCR chimerism, the artificial formation of cross-overs between the two variants during PCR amplification, is one potential explanation for the additional haplotype.

### Reference alignment bias explains the relative paucity of the fourth *RET* haplotype

If PCR chimerism was the explanation for the extra haplotype then we would have expected all four haplotypes to be present at high, and approximately equal, frequency. However, we noticed that the haplotype with the highest frequency had reference allele bases at both mutation positions and the haplotype with variant bases at both mutation positions was the haplotype at very low frequency. Therefore, one possibility to explain the relative absence of the fourth haplotype is reference alignment bias. To test this we re-aligned the reads, but this time we changed the reference bases to the variant base at the mutation positions. This completely reversed the frequency of the haplotypes, such that the previously lowest frequency haplotype was now the highest frequency haplotype and *vice versa*. This strongly supports the argument that a combination of PCR chimerism and reference alignment produced the pattern of haplotypes we observed (Table 2B).

### Pacific Biosciences sequencing confirms all four haplotypes are present in the sequenced library

**Figure 2:**
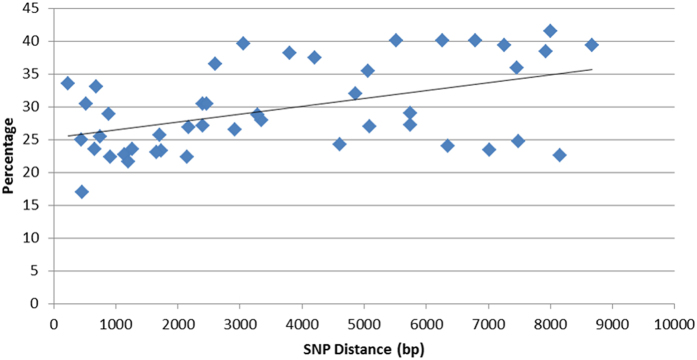
Distance between SNPs within the sequenced region versus abundance of the 3^rd^ and 4^th^ (chimeric) haplotypes. The line shows a positive linear correlation between the distance apart of the SNPs and the abundance of the 3^rd^ and 4^th^ haplotypes.

ONT sequencing suffers from a high per base error rate and this may have affected our results. We therefore sequenced the same *RET* amplicon used in our ONT analyses using the PacBio platform where sequencing quality is substantially higher (Table 1). All four haplotypes were present this time, although a more subtle reference alignment bias was still detected (Table 2A).To confirm that the four haplotypes were a result of chimerism we looked at other SNPs closer together within the sequenced region. These show high levels of a third and fourth haplotype and there is a positive correlation between the distance between variants and the proportion of the third and fourth haplotype (Speaman’s rho = 0.44, *P *= 0.003, Fig. 2).

### PCR chimerism and reference alignment bias provides an alternative explanation of the results of Ammar *et al*.

Ammar *et al.*^4^ used reads from the ONT MinION to determine *CYP2D6* haplotypes in a single individual. However, they detected strong abundance of a third haplotype which was not predicted from their other methods such as statistical phasing. They suggested this third haplotype could have arisen due to template switching or sample contamination. We re-analysed their data and believe their results are best explained by the same phenomena that we observed in our *RET* phasing data.

Using the aligned BAM file provided by Ammar *et al.*^4^ we can show that all four haplotypes are present in the sample, but that the fourth haplotype is masked by reference bias in the alignment in their analyses (Table 3A,B). Therefore, the Ammar *et al.*^4^ results are best explained by chimeric PCR products in combination with the effect of reference bias in the alignment. It is also possible that reference alignment bias alone could account for the unexpected third haplotype given that it is the reference haplotype. This means that from their ONT MinION data alone the true haplotypes cannot be determined.

### Reducing PCR cycles results in lower incidence of chimerism

Single molecule sequencing from PCR carried out with a reduced number of cycles provides evidence that the two mutations investigated (p.R418X and p.L56M) are in *trans* and thus both mutations might contribute to the disease in our patient. When we repeated the PCR amplification of the *RET* product using a new set of primers (see methods) and different numbers of cycles it becomes clear that the incidence of chimeras increases with the number of cycles. When the product of 39 cycles is sequenced on the PacBio there are four haplotypes present at nearly equal levels whereas at 29 cycles only 6.5% of reads are from chimeric haplotypes (Table 4). Further studies are needed to develop the statistical framework for generating precise probabilities of each haplotype being a true haplotype that appropriately takes into account the various potential biases, for example reference alignment bias. At a simple level, however, 93% of the reads from the 29-cycle experiment supported the first two haplotypes, and one simple approach to this problem is to call the first two haplotypes as the true ones when more than, for example, 90% of reads consist of the first two haplotypes. Using this metric we can then be >99.9% confident (using the binomial distribution) that the true haplotypes in this case are the first two haplotypes. Even at 29 cycles chimeras are likely the largest source of error in the results, as only 0.6% of reads give haplotypes not formed from the two known bases at each position; these likely arise from sequencing error. This also allows us to estimate that the vast majority of the third and fourth haplotypes are due to chimera formation as opposed to sequencing error (6.5%*0.6% = 6.46%). PCR carried out with fewer than 29 cycles did not yield enough product for sequencing, this means that a reduction of cycles alone will not be able to entirely eliminate chimera formation.

## Discussion

We have highlighted issues with phasing variants using long-read sequencing technologies. PCR chimera formation and reference alignment bias issues can prevent the successful phasing of variants using long-read sequencers.

### Chimeric molecules are a common PCR artefact

Chimeras are a common source of error during PCR, where template switching can result in recombinant molecules^10^. It is a known common artefact in amplicon bases studies of microbial diversity^11^ and has been reported as a major source of erroneous sequences in 16S reference databases^12^. The rates of PCR chimeras will vary based on PCR conditions and target products. However, it has been suggested that more than 45% of reads in some sequencing datasets can be of chimeric origin^11^,^12^,^13^. While experimental measurements of chimera formation during PCR have estimated that greater than 30% of the final products will be chimeric^14^. The formation of chimeric products during PCR has been previously reported in phasing studies^15^. Chimera formation is likely to be especially problematic when generating long amplicons, where template switching is more likely to generate chimeric products. In contrast to studies of microbial diversity where tools have been developed specifically to filter out chimeric reads^16^,^17^, phasing studies do not have a clear method of filtering out chimeric reads as they cannot assume the two parent sequences will be significantly dissimilar. As demonstrated in our study, chimeric reads can confound the ability to successfully call haplotypes.

We demonstrate that the frequency of chimeric molecules being produced can be reduced by performing fewer PCR cycles, as the production of chimeras occurs at a lower rate during the exponential phase of the PCR than at the plateau phase^15^. However this reduces the amount starting product can be amplified by, meaning that a larger amount of starting DNA will be required for sequencing. Alternative methods for isolating the required amplicon such as targeted capture could offer a PCR-free way to generate sequencing libraries. Another option is to use droplet PCR^18^ to amplify molecules in isolation and avoid the problem of chimeric reads.

### Reference bias occurs in the presence of a high insertion and deletion rate

In both our results and those of Ammar *et al.*^4^, the presence of chimeric reads was masked by reference bias during alignment. Reference bias is a problem known to occur when there is a high rate of insertions and deletions in the sequencing. In order to get reads with such errors to align, the gap opening penalty is reduced in order to maximise alignment performance. However this can result in reference bias when the aligner hides a true mismatch inside an insertion to give a better scoring alignment^19^. Reference bias has been shown to occur with PacBio sequencing, and based on reported rates of indels for the ONT MinION it can be inferred as a likely artefact in that data as well. While chimeric PCR products are an issue with library preparation rather than the sequencing, the reference alignment bias observed in this study is likely to be partly a factor of the high insertion and deletion rate of the sequencing technologies. Thus all studies where the calling of a specific base is important must take care when using these technologies.

Alignment bias will be reduced as the error rate in the sequencing technologies improves. Methods such as local realignment could solve this issue. However such programmes are currently designed to use information from all the aligned reads to correct for alignment bias around a real insertion against the reference^20^, rather than to correct insertion errors in the individual reads.

### The results of Ammar *et al*. are best explained by chimeric reads and reference bias

We have highlighted issues with phasing variants using long read sequencing technologies. We question the explanation that the unexpected haplotypes in the ONT MinION data from Ammar *et al.*^4^ could be due to sample contamination. We know that this is unlikely to be the explanation for our results as within our amplicon there are two SNPs, 457 bp apart, at positions 43108014 (rs72781236) and 43108471 (rs72781237) that have four clear haplotypes despite being perfectly correlated (r2 = 1; D’ = 1) within the population (i.e. they form only two haplotypes) in the >1000 individuals sequenced in the 1000 genomes projects^21^. Having observed the same patterns in the Ammar *et al.*^4^ data we find it a more parsimonious explanation that they encountered the same issues we have highlighted.

Single molecule sequencing offers the promise of phasing mutations across great distances directly from the reads. However we have shown the pitfalls currently inherent in these methods. Formation of chimeric molecules in the PCR amplification step can render it impossible to call the true haplotypes. The ideal solution would be PCR-free library generation such as targeted capture and such methods are being developed^22^, although this approach is relatively time-consuming and costly compared to PCR. Using fewer PCR cycles will greatly reduce these artefacts. It is not possible to provide exact recommendations because of differences between experiments in target fragments and optimal PCR conditions, but our advice is that researchers should use the fewest PCR cycles that provide sufficient product for sequencing. Even then a substantial fraction of reads may be chimeric, but as we show, it is still possible to assign true haplotypes with some confidence in the presence of low levels of chimeras. However, phasing results can also be clouded by reference bias in the alignment caused by the current high insertion and deletion rates of the long read sequencing technologies. As error rates from long read sequencers reduce, this will become much less of an issue. However, researchers should be aware of this problem and if their results appear to reflect the current reference bases should consider repeating the alignment with SNP bases reversed in the reference, as done in this study, to assess the impact of alignment bias on their results. A combination of method refinement and continued improvements in the sequencing technologies should result in the ability to correctly phase long range mutations directly from single molecule sequencing reads, but our data and re-analysis of the data from Ammar *et al.*^4^ shows the potential for misleading conclusions when these factors are not considered.

## Methods

### PCR amplification of the *RET* product

Long-range PCR was carried out using the SequalPrep Long PCR kit with dNTPs (Life Technologies), using 45 ng of template DNA per 20 μl reaction under conditions as recommended by the kit manufacturer. All reactions used the same forward primer (RET-f, 5′-AAGCAGTTCTTTTCTAGCCCGTGTG-3′) and either reverse primer RET-r1 (5′-AGTGTCACCTGCCTCCCTGTG-3′) or RET-r2 (5′-CCAGTCTACTCTGTGCTGGTTGGG-3′) which amplify genomic targets of 9028 bp or 9061 bp respectively. For reactions using primers RET-f/RET-r1, amplification was carried out for a total of 39 cycles; using RET-f/RET-r2, amplification was for 39, 34 or 29 cycles as indicated. After amplification, the 9 kb full-length products were purified and analysed using either a Bioanalyzer 2100 or TapeStation 2200 system (Agilent Technologies) prior to preparation of sequencing libraries.

### Construction and sequencing of ONT MinION library

The *RET* library was initially sequenced on the R6 MinION chemistry then re-sequenced on R7.3. The library preparation followed the manufacturer’s instructions (SQK-MAP001 for R6 and SQK-MAP005 for R7.3) except where specified in Supplementary material 1.

### Construction and sequencing of SMRTbell library

The SMRTbell templates were constructed from PCR products (1.4 −8.5 ug) using the DNA Template Prep Kit 1.0 (Pacific Biosciences, Menlo Park, CA). For the PacBio1 template the 10 kb low-input protocol was used following manufacturer instructions. For PacBio C29–C36 the 20 kb template preparation protocol was used, with the following modifications: DNA purifications were performed with (0.6×) Agencourt AMPure PB beads and 30 minutes incubations on a rotator. Size-selections were carried out with BluePippin on 0.75% dye free agarose gels (Sage Science) and template size was estimated with Agilent Tapestation or Bioanalyzer. Binding calculation (MagBead OCPW), primer annealing, P6v2 DNA polymerase binding and MagBead binding was carried out according to manufacturer’s instructions (template preparation guide V26). Briefly, sequencing primers were annealed to either 1.28 nM or 0.5 nM template at a final concentration of 0.833 nM (1:20 ratio) by denaturing the primer at 80 °C for 2 minutes, cooling to 4 °C before incubating with the library at 20 °C for 30 minutes following instructions in the complex setup and loading calculator provided by the manufacturer. Annealed templates were either stored at −20 °C until or directly used for polymerase binding. DNA polymerase enzymes were stably bound to the primed sites of the annealed SMRTbell template using the DNA Polymerase Binding Kit (PacBio). SMRTbell templates were incubated with 0.5 nM of P6v2 DNA polymerase in the presence of phospholinked nucleotides at 30 °C for 30 minutes. SMRT Sequencing was performed on three SMRT cells for C34 and two SMRT cells for PacBio1, C29 and C39. Sequencing movie collection was performed on the PacBioRS2 for either 4 hours (PacBio1) or 6 hours.

### Bioinformatic analyses

Reads from the ONT MinION were aligned with LAST^7^ against the human genome (build GRCh38) plus the sequence of the Lambda control spike and using only the best scoring alignment for each read. For PacBio data we selected all reads of insert with reads lengths of 8600–9200 bp. These were aligned with BLASR^23^ against the human genome (build GRCh38) and using only the best scoring alignment for each read. Alignment and error rates are shown in Table 1. The error rate was calculated from aligned reads as a ratio of total edit distance (the number of base changes required to go from the reference sequence to the read) to the number of mapped bases. A sensitivity analysis on the PacBio data showed that using only higher quality reads (those ROI generated from 5 or more subreads) did not change the result. Reads were phased on the two mutations we targeted, 43100551 C > A and base 43109219 C > T (build GRCh38).When re-analysing the data from Ammar *et al.*^4^ we used their aligned BAM file which they generated using BLASR^23^ to align their reads against the human genome (build GRCh37). When phasing *CYP2D6* reads we required alignment at all 9 SNP bases but only called haplotypes from 2 positions, as recommended by their methods plus personal communications.

To test the effect of reference bias in the alignment we conducted an experiment where we changed the reference bases to the variant base at the mutation/SNP positions. Single base changes were made at both SNP positions under analysis to make the reference the alternate base at those positons. For our *RET* data this consisted of changing base 43100551 C > A and base 43109219 C > T (build GRCh38). For the Ammar *et al.*^4^ *CYP2D6* data this consisted of changing base 42524947 C > T and deleting the T base at 42524244 (build GRCh37). When measuring the haplotypes against the altered reference for Ammar *et al.*^4^ *CYP2D6* data a T was recorded as being present in the read at position 42524244 if the base was G (the following reference base in the original reference) with a T insertion, while a G with no insertion was interpreted as a deletion in the read.

## Additional Information

**How to cite this article**: Laver, T. W. *et al.* Pitfalls of haplotype phasing from amplicon-based long-read sequencing. *Sci. Rep.* **6**, 21746; doi: 10.1038/srep21746 (2016).

## Supplementary Material

Supplementary Information

## Figures and Tables

**Table 1:** Sequencing data statistics.

	Total reads analysed	Reads aligned	Error rate (%)	Reads aligned to both snps
MinION1	6915	1812	37.6	111
MinION2	4365	1833	34.2	81
PacBio1	3459	2885	21.4*	261
PacBio C29	41244	20602	3.97*	15200
PacBio C34	54294	27147	3.74*	21945
PacBio C39	26722	13361	3.85*	11683

*These are based on ROI (read of insert) error rates.

**Table 2A:** *RET* haplotypes.

	CC (ref/ref)	CT (ref/alt)	AC (alt/ref)	AT (alt/alt)	Total other haplotypes
MinION1	36 (32.4%)	25 (22.5%)	16 (14.4%)	3 (2.7%)	31 (27.9%)
MinION2	21 (25.9%)	21 (25.9%)	6 (7.4%)	7 (8.6%)	26 (32.1%)
PacBio1	84 (32.2%)	67 (25.7%)	59 (22.6%)	39 (14.9%)	12 (4.6%)

**Table 2B:** *RET* haplotypes with reference bases reversed.

	CC (alt/alt)	CT (alt/ref)	AC (ref/alt)	AT (ref/ref)	Total other haplotypes
MinION1	2 (1.8%)	22 (19.8%)	14 (12.6%)	34 (30.6%)	39 (35.1%)
MinION2	3 (4.9%)	16 (26.2%)	8 (13.1%)	13 (21.3%)	21 (34.4%)
PacBio1	28 (12.6%)	54 (24.3%)	58 (26.1%)	74 (33.3%)	8 (3.6%)

**Table 3A:** Ammar *et al. CYP2D6* haplotypes.

	CT (ref/ref)	C- (ref/alt)	TT (alt/ref)	T- (alt/alt)	Total other haplotypes
Original reference	86 (33.2%)	61 (23.6%)	52 (20.1%)	6 (2.3%)	54 (20.9%)

**Table 3B:** Ammar *et al. CYP2D6* haplotypes with reference bases reversed.

	CT (alt/alt)	C- (alt/ref)	TT (ref/alt)	T- (ref/ref)	Total other haplotypes
Reversed reference	1 (0.4%)	42 (18.0%)	44 (18.9%)	87 (37.3%)	59 (25.3%)

**Table 4:** *RET* reduced PCR haplotypes.

	CC (ref/ref)	CT (ref/alt)	AC (alt/ref)	AT (alt/alt)	Total other haplotypes
PacBio C29	632 (4.2%)	7104 (46.7%)	7021 (46.2%)	356 (2.3%)	87 (0.6%)
PacBio C34	3860 (17.6%)	7073 (32.2%)	7556 (34.4%)	3284 (15.0%)	172 (0.8%)
PacBio C39	2552 (21.8%)	3326 (28.5%)	3424 (29.3%)	2281 (19.5%)	100 (0.9%)
