# Supplementary material for: Pitfalls of haplotype phasing from amplicon-based long-read sequencing
Source: Sci Rep. 2016 Feb 17;6:21746. doi: 10.1038/srep21746 (PMC4756330; doi:10.1038/srep21746)
Supplement: Supplementary Information [file srep21746-s1.pdf]

Pitfalls of haplotype phasing from amplicon-based long-read sequencing

Thomas W. Laver, Richard C. Caswell, Karen A. Moore, Jeremie Poschmann, Matthew B. Johnson, Martina M. Owens, Sian Ellard, Konrad H. Paszkiewicz, Michael N. Weedon

Supplementary material

Supplementary material 1

The MinION library preparation protocol was adapted from ONT genomic DNA sequencing protocol (SQK-MAP001) version MN001_1115_revC. 5 µl lambda phage spike-in DNA (CS, ONT) was added to 1 µg CP-*RET* PCR amplified DNA. Fragments were end-repaired and adenylated using NEXTflex Rapid DNAseq kit (B100 Scientific), incubated at 20°C for 20 minutes, 72° for 20 min and cooled to room temperature. The reaction was purified using an equal volume of Ampure XP beads and eluted in 53ul 1x a-tailing buffer (NEB). 392 ng DNA was ligated to Adapter mix (ONT) and HP adapter (ONT) for 10 minutes at 20°C and purified using 0.6 vol Ampure XP beads. The beads were washed with wash buffer (ONT) and eluted in 25 µl elution buffer (ONT). The tether was incubated with the adapter-ligated DNA for 10 minutes before conditioning which involved adding the HP motor and continuing the incubation at room temperature overnight. This pre-sequencing mix was stored briefly on ice. After Platform _QC a flowcell was conditioned by incubating the chamber twice with 150 µl EP for 10 minutes. Immediately before sequencing, 6 µl pre-sequencing mix was added to a 140 µl EP buffer (ONT) and 4 µl fuel mix (ONT) and mixed very gently before being loaded on to the MinION flowcell and sequenced using MAP_48h_Sequencing_Run.py and MinKNOW software.

For the second sequencing run the ONT protocol SQK-MAP005 was followed unless indicated and all reactions carried out at room temperature. 1.26 µg CP-RET PCR products were repaired using PreCR treatment (NEB) at 37°C for 30 min and purified using an equal volume of AmpureXP beads (Beckmann Coulter). 5 µl lambda phage spike-in DNA (CS, ONT) was added to 30 µl repaired DNA. Fragments were end-repaired and adenylated using NEXTflex Rapid DNAseq kit (B100 Scientific), incubated at 20°C for 20 minutes, 72° for 20 min and cooled to room temperature. Adapter mix (AMX, ONT) and HP adapter (HPA, ONT) were ligated to the adenylated DNA using the NextFlex Rapid ligase mix incubated at room temperature for 10 min. The library was purified using His-beads (Invitrogen) using the bead binding buffer and bead washing buffer provided in the SQK-MAP005 library preparation kit. 113 ng DNA was eluted in 25 µl elution buffer (ELB, ONT). This pre-sequencing mix (library) was stored briefly on ice during Platform_QC and flowcell priming. Immediately before sequencing, 6 µl pre-sequencing mix was added to a mixture of 75 µl 2x running buffer (RNB, ONT), 66 µl nuclease-free water and 3 µl fuel mix (FMX,ONT) and mixed very gently before being loaded on to the MinION flowcell and sequenced using MAP_48h_Sequencing_Run.py and MinKNOW software version 0.50.1.15 B201505221054.
